# Pulse oximetry-based capillary refilling evaluation predicts postoperative outcomes in liver transplantation: a prospective observational cohort study

**DOI:** 10.1186/s12871-020-01171-y

**Published:** 2020-09-29

**Authors:** Miyuki Yamamoto, Kent Doi, Naoki Hayase, Toshifumi Asada, Nobuhisa Akamatsu, Junichi Kaneko, Kiyoshi Hasegawa, Naoto Morimura

**Affiliations:** 1 Department of Acute Medicine, The University of Tokyo, 7-3-1 Hongo, Bunkyo-ku, Tokyo, 113-0033 Japan; 2 Hepato-Biliary-Pancreatic Surgery Division, Artificial Organ and Transplantation Division, Department of Surgery, The University of Tokyo, Tokyo, Japan

**Keywords:** Capillary refill time, Non-invasive, Tissue perfusion, Liver transplantation, Perioperative management, Pulse oximeter

## Abstract

**Background:**

Capillary refill time (CRT) is a non-invasive technique to evaluate tissue perfusion, and quantitative CRT (Q-CRT) adapted to pulse oximetry was developed with patients with sepsis and compared to blood lactate and sepsis scores. In post liver transplantation, large amounts of fluid administration are necessary for maintaining tissue perfusion to grafted liver against intravascular hypovolemia. This study aimed to evaluate whether Q-CRT can predict poor outcomes by detecting peripheral tissue perfusion abnormality in patients with liver transplantations who were treated with massive fluid administration.

**Methods:**

In this single-center prospective cohort study, we enrolled adult patients with liver transplantations between June 2018 and July 2019. Measurement of Q-CRT was conducted at intensive care units (ICU) admission and postoperative day 1 (POD1).

**Results:**

A total of 33 patients with liver transplantations were enrolled. Significant correlations of Q-CRT and ΔA_b_, a tissue oxygen delivery parameter calculated by pulse oximetry data, at ICU admission with the postoperative outcomes such as length of ICU and hospital stay and total amount of ascitic fluid discharge were observed. Quantitative CRT and ΔA_b_ at ICU admission were significantly associated with these postoperative outcomes, even after adjusting preoperative and operative factors (MELD score and bleeding volume, respectively). However, quantitative CRT and ΔA_b_ at POD1 and changes from ICU admission to POD1 failed to show significant associations.

**Conclusions:**

Q-CRT values were significantly associated with postoperative outcomes in liver transplantation. Although the mechanisms of this association need to be clarified further, Q-CRT may enable identification of high-risk patients that need intensive postoperative managements.

## Background

Monitoring tissue perfusion is important for the management of critically ill patients in intensive care units (ICUs), since insufficient oxygen delivery to peripheral tissues is strongly associated with organ dysfunction in many conditions such as sepsis and postsurgical organ failure [1–3]. In contrast to hemodynamic parameters that can be measured by pulmonary artery catheters and ultrasound techniques, only few measurements that can evaluate tissue perfusion are clinically available. Sublingual videomicroscopy can measure tissue perfusion in a real-time manner; however, these devices are expensive and not easily applied to clinical use [4, 5].

Capillary refill time (CRT) is a simple, fast, and non-invasive method for evaluating tissue perfusion. This method has been used in the field of disaster medicine as a triage tool [6, 7]. A recent clinical trial demonstrated that inclusion of CRT measurement in septic shock management at ICUs can reduce mortality risk [8]. Although CRT may be affected by inter-examiner differences [9, 10], a recently developed device can measure CRT quantitatively by using a pulse oximeter [11]. Our recent validation studies discovered a significant correlation between venous blood lactate levels and quantitative CRT (Q-CRT), as measured via the pulse oximeter-based device, in a cohort of ICU patients and emergency department (ED) patients [11, 12]. Q-CRT in 23 ICU patients was demonstrated to be statistically correlated with lactate levels (Spearman’s rank correlation coefficient, 0.681; *p* < 0.001) [11]. In addition to Q-CRT, we developed the delta A_b_ (ΔA_b_) measure, which is based on the amount of light absorbed into finger tissue and blood flow by subtracting the light quantity input value from the output value. ΔA_b_ is assumed to reflect overall peripheral oxygen delivery and integrates blood flow, oxygenation, and hemoglobin concentration [12].

Liver transplantation is widely performed for end-stage liver failure. Advances in surgical procedures and immunosuppressive therapy significantly improved the outcomes of liver transplantation [13–15]. Postsurgical management of liver transplantation is of great importance in critical care medicine, because large amounts of fluid administration is frequently necessary to maintain intravascular volume and adequate portal vein blood flow to the grafted liver. Although the blood lactate level is utilized to evaluate oxygen delivery to peripheral tissues in managing septic shock, alterations in lactate levels are influenced by factors other than peripheral hypoperfusion, including liver dysfunction [16, 17]. Several studies reported on the benefit of lactate reduction as a clinical parameter for monitoring early graft function following liver transplantation [18–20]. So, far, no study has assessed the clinical application of Q-CRT in liver transplantation, although we previously evaluated Q-CRT in septic patients. This prospective observational study was performed in order to examine whether Q-CRT and ΔA_b_ values measured following liver transplantation are associated with postoperative outcomes.

## Methods

### Patient population and study design

In this single-center prospective cohort study, we enrolled patients who received liver transplantation and who were admitted to the ICU of the University of Tokyo Hospital between June 2018 and July 2019. Orthotopic liver transplantation followed the standardized procedure in all cases with the living donor partial liver or the deceased donor whole liver. Intraoperative porto-caval shunt was never created in this series. All 18 years old or older patients were eligible, and patients who did not agree to participate and/or who demonstrated missing data were excluded. The study protocol adhered to the Declaration of Helsinki and was approved by the institutional review board of the University of Tokyo. Informed consent was obtained from each participant.

Regarding postoperative management, chest and abdominal radiographs, and abdominal and cardiac ultrasonography examinations were performed at least daily until POD14. Treatment with diuretics such as furosemide, spironolactone, and human atrial natriuretic peptide, albumin preparations, and vasopressors was administered at the physician’s clinical discretion.

### Q-CRT and ΔA_b_ measurements

Measurements of Q-CRT and ΔA_b_ were performed based on the principle of pulse oximetry. The measurement principle and device wearing method are described in detail, in our previous report [12]. Briefly, transmitted light quantity was measured by a pulse oximeter (OLV-3100, Nihon Kohden Corporation, Tokyo, Japan) equipped with an SpO_2_ sensor. This device has not been approved for clinical use yet. Mechanical pressure with 500 mmHg lasting for 5 s was applied to the patient’s nail bed of the index or middle finger. The values of Q-CRT and ΔA_b_ were determined as the average of five measurements. The ICU room temperature was kept between 24 °C and 26 °C, and the room lighting was on during the measurement.

Quantitative CRT was defined as the time in seconds from the release of the pressure to the time when the blood flow reached 90% of the original flow, which was measured for 5 s at the beginning of the test before applying pressure. Transmitted light quantity measured by a pulse oximeter is equivalent to the amount of light absorbed into finger tissue and blood flow and equivalent to the amount of light absorbed into finger tissue only under compression. The quantity of light dimmed by blood only defines the ΔA_b_ (delta A_b_), which is the difference between the quantity of light dimmed under infrared light and that dimmed under red light. In short, ΔA_b_ can be determined by oxidized hemoglobin levels (oxygen saturation), hemoglobin concentration, and tissue thickness with blood flow (peripheral circulation blood volume) [12].

### Data collection

The following patient characteristics and clinical data were collected from the medical records: age, sex, body temperature, height, weight (before transplantation), amount of ascites, and underlying liver disease. Preoperative model for end-stage liver disease (MELD) and Child–Pugh scores were calculated. Donor information such as age, sex, weight, and graft size were collected. The following clinical variables were evaluated as intraoperative factors: operative time, bleeding volume, intraoperative fluid balance, anhepatic time, and ischemic time of graft.

At ICU admission after liver transplantation surgery, Q-CRT and ΔA_b_ measurements were performed. Other clinical parameters, including vital signs, usage of vasoactive agents, and measurements of central venous pressure (CVP), were also obtained. Additionally, laboratory data recorded at ICU admission included blood lactate, hemoglobin (Hb), total bilirubin, and prothrombin time international normalized ratio (PT-INR). We performed hepatic blood flow assessment twice daily, determining the amount of fluid volume based on the results of echocardiography and CVP monitoring. Q-CRT, ΔA_b_, and blood lactate were also measured at postoperative day 1 (POD1).

Daily ascites was defined as the total amount of ascites through the abdominal tube and exudate. Abdominal drain tubes were routinely inserted near the surface of the donor graft, behind the graft hilum, and into the rectovesicular (Douglas) pouch. In cases with simultaneous splenectomy, an additional drain tube was placed into the left sub-phrenic cavity. In patients with pleural effusion, drainage was performed by the placement of thoracic catheter. The total amount of discharge was recorded, including pleural effusion.

### Sample size estimation

As a small pilot study, we compared the Q-CRT at ICU admission between five liver transplant patients with massive ascites (ascites volume > 1000 ml/day on POD 14) and five liver transplant patients with non-massive ascites. The estimated difference of the mean and standard deviation were 0.55 and 0.41, respectively, using log-transformed data. On this basis, the sample size was calculated as being 10 patients with massive ascites and 10 patients with non-massive ascites, assuming a type I error rate of 0.05, a power of 0.8, an anticipated effect size d = difference of means/ standard deviation = 1.34.

### Statistical analysis

Continuous variables were presented as medians with interquartile ranges, and categorical variables were presented as percentages. Categorical data were compared by the chi-square or Fisher’s exact test, while continuous data were compared by Student’s *t* test or Wilcoxon’s rank-sum test. Correlations between variables were analyzed using Spearman’s rank correlation. All continuous parameters with a skewed distribution were entered into these models as log-transformed variables using the natural logarithm to the base e. A multivariable logistic regression model was used to evaluate the independent contribution of Q-CRT or ΔA_b_ to the outcomes by adjusting predefined preoperative (MELD score) and operative (blood loss during surgery) factors. The cut-off point was determined using receiver operating characteristic (ROC) analysis with Youden-index. All statistical analyses and calculations were performed using the JMP® Pro software (version 14.2.0; SAS Institute, Cary, NC). A two-tailed probability (p) value < 0.05 was considered statistically significant for all tests.

## Results

### Patient characteristics

We enrolled a total of 33 patients with liver transplantations. Table 1 shows characteristics and clinical parameters at ICU admission after surgery. Hyperlactemia and hyperbilirubinemia were observed. Additional file 1 summarized the preoperative baseline characteristics, graft conditions, surgery-related factors of the study population, and other clinical parameters at ICU admission after surgery.

**Table 1:** Characteristics and clinical parameters at ICU admission

Variables		
Recipient characteristics	age (years)	52 (43–60)
	male sex	17 (51.5%)
	body weight (kg)	57.7 (53.3–68.0)
	Child–Pugh score	10 (8–12)
	MELD score	15 (10–19)
	perioperative ascites	13 (39.4%)
Clinical parameters at ICU admission	body temperature (°C)	37.2 (36.6–37.6)
	heart rate (/min)	101 (95–109)
	mean arterial pressure (mmHg)	73 (66–83)
	usage of vasoactive agents	11 (33.3%)
	lactate (mg/dL)	5.4 (2.9–9.1)
	total bilirubin (mg/dL)	3.1 (2.2–6.0)
	PT-INR	1.37 (1.26–1.50)
	portal venous flow velocity (cm/sec)	54.8 (37.5–82.1)

Summary statistics are reported as No. (%), medians (lower and upper quartiles)

*MELD* the model for end-stage liver disease, *PT-INR* prothrombin time international normalized ratio

### Q-CRT and ΔA_b_ at ICU admission

**Fig. 1:**
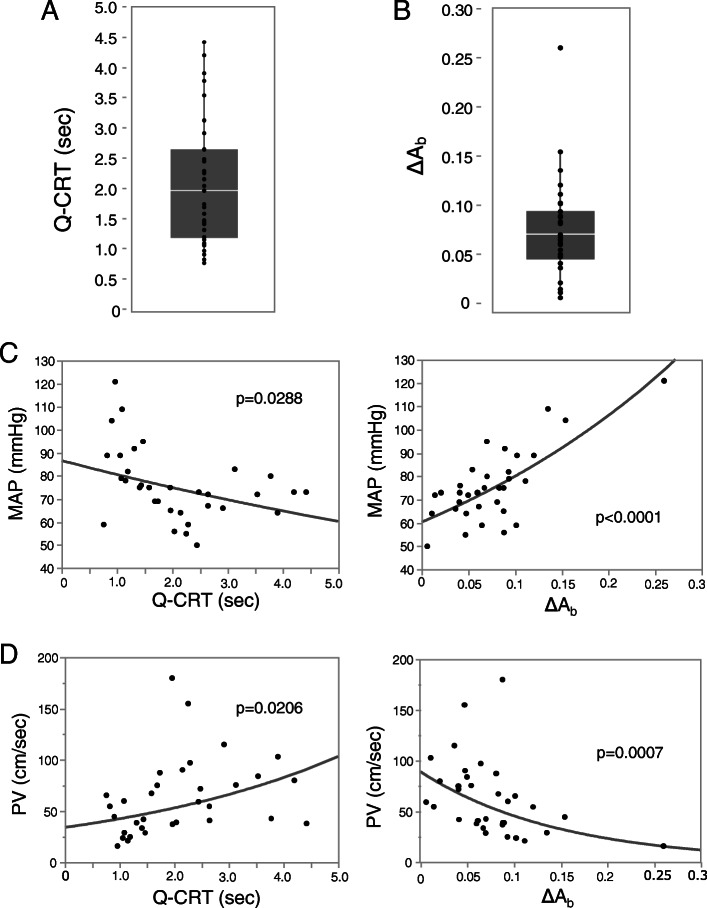
Quantitative capillary refill time and ΔA_b_ at ICU admission. Distributions of Q-CRT (**a**) and ΔA_b_ (**b**) measured at ICU admission. Box plots show the median (center line), the 25th and 75th percentiles, and the range. Correlations of Q-CRT and ΔA_b_ with mean arterial pressure (MAP) (**c**) and portal venous velocity (PV) (**d**). Measures observed at similar time points (*N* = 33). The *p* value is indicated in each graph

Figure 1a and b show the values of Q-CRT and ΔA_b_ at ICU admission, respectively. Significant correlations were observed for Q-CRT and ΔA_b_ with regard to mean arterial pressure (MAP) (Fig. 1c), portal vein (PV) velocity (Fig. 1d), while no significant correlation of Q-CRT or ΔA_b_ with blood lactate, heart rate (HR), CVP, hemoglobin, and hepatic artery (HA) velocity was observed (Additional file 2).

### Postoperative data

Four patients developed early allograft dysfunction (EAD) [21], and one patient had graft failure 6 months following transplantation. The Q-CRT and ΔA_b_ values showed no difference between the EAD and the non-EAD patients (data not shown). One year later, all patients were still alive.

**Fig. 2:**
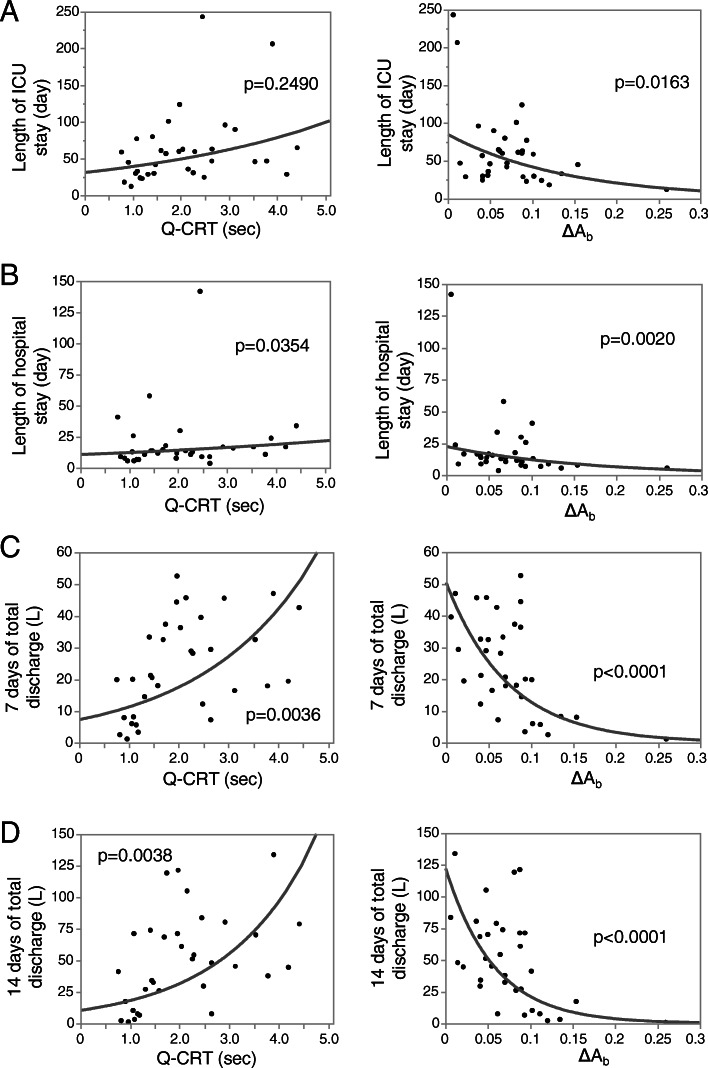
Correlations of Quantitative capillary refill time, ΔA_b_ with the outcomes. Correlations with length of post-surgery ICU stay (**a**), hospital stay (**b**), the total amount of discharge for 7 days (**c**) and 14 days (**d**) after the surgery are shown (*N* = 33). The *p* value is indicated in each graph

The median lengths of ICU and hospital stay were 13 days (IQR 9–18) and 47 days (IQR 30–71), respectively. With regard to the length of ICU stay and post-surgery hospitalization, significant correlations were observed for both Q-CRT and ΔA_b_ (Fig. 2a-b). The median total ascitic discharge at 7 and 14 days was 20.6 L (IQR 10.3–36.9) and 45.4 L (IQR 21.9–72.7), respectively. Both Q-CRT and ΔA_b_ showed significant correlations with the total amount of ascites discharge 7 and 14 days after the surgery (Fig. 2c-d). Portal vein, not hepatic artery and vein, blood flow measured just after the surgery demonstrated significant correlation with ascites discharge 7 and 14 days after surgery (Additional file 3).

Based on the preoperative ascites volume, the patients were divided into three groups (a no ascites group, a non-massive ascites group: 1–999 ml, and a massive ascites group: > 1000 ml). Patients who had preoperative massive ascites displayed the largest amount of postoperative ascites (Additional file 4). The enrolled patients were divided into two groups using a cutoff value of the total amount of ascitic discharge of 1000 mL/day at POD14, based on the diagnostic criteria for small-for-size syndrome and previous reports [22–24] (Table 2 and Additional file 5). The group exhibiting more ascitic discharge showed significantly longer Q-CRT and lower ΔA_b_ values at ICU admission. The Q-CRT and ΔA_b_ cutoff values, the area under the curve (AUC), and the 95% confidence interval (CI) for each outcome in patients with massive ascites are shown (Additional file 6). Multiple regression analysis demonstrated that Q-CRT and ΔA_b_ at ICU admission were significantly associated with the length of ICU stay, length of post-surgery hospitalization, and total amount of ascitic discharge for 14 days after surgery, even after adjusting confounding factors of preoperative MELD score and intraoperative blood loss (Table 3).

**Table 2:** Comparison of the non-massive ascites group with the massive ascites group

		Non-massive ascites group (*N* = 10)	Massive ascites group (*N* = 23)	*p* value
Recipient characteristics	age (years)	45 (34–58)	54 (47–62)	0.1580
	male sex	5 (50%)	12 (36.4%)	0.9086
	body weight (kg)	67.5 (57.2–71.8)	54.6 (52.0–67.0)	0.0525
	Child–Pugh score	8 (6–10)	10 (9–12)	0.0089
	MELD score	13 (8–16)	16 (10–24)	0.0593
	preoperative ascites	1 (10%)	12 (36.4%)	0.0227
Clinical parameters at ICU admission	body temperature (°C)	37.5 (37.2–37.8)	37.0 (36.5–37.5)	0.0544
	heart rate (/min)	98 (93–102)	102 (95–116)	0.1580
	mean arterial pressure (mmHg)	86 (76–99)	72 (64–75)	0.0025
	usage of vasoactive agents	1 (10%)	10 (43.5%)	0.0608
	lactate (mmol/L)	4.7 (1.9–9.0)	5.4 (3.2–9.5)	0.4929
	total bilirubin (mg/dL)	2.4 (1.1–3.8)	3.2 (2.4–6.7)	0.0848
	PT-INR	1.32 (1.22–1.43)	1.37 (1.27–1.50)	0.4804
	Q-CRT (sec)	1.17 (1.03–1.49)	2.25 (1.68–3.12)	0.0061
	ΔA_b_	0.097 (0.067–0.124)	0.060 (0.040–0.088)	0.0109
	early allograft dysfunction	1 (10%)	3 (13%)	0.8055
Outcomes	Length of ICU stay (day)	8 (6–13)	16 (11–26)	0.0025
	Length of hospital stay (day)	30 (22–47)	60 (45–90)	0.0040
	7 days total discharge (L)	6.7 (3.3–18.7)	32.6 (19.5–42.6)	0.0003
	14 days total discharge (L)	7.8 (3.3–27.8)	68.7 (44.6–80.5)	< 0.0001

Summary statistics are reported as No. (%), medians (lower and upper quartiles)

*MELD* model for end-stage liver disease, *PT-INR* prothrombin time international normalized ratio

**Table 3:** Multiple regression analysis of Q-CRT and ΔA_b_ at ICU admission

	ICU Stay		Hospital Stay		Ascitic discharge	
Variable	odds ratio (95% CI)	*p* value	odds ratio (95% CI)	*p* value	odds ratio (95% CI)	*p* value
MELD score	0.935 (0.817–1.052)	0.2660	0.870 (0.759–0.996)	0.0222	0.770 (0.588–1.008)	0.0075
blood loss	1.000 (0.999–1.000)	0.3418	1.000 (0.999–1.000)	0.0753	1.000 (0.999–1.000)	0.8759
Q-CRT	0.538 (0.212–1.182)	0.1256	0.304 (0.106–0.871)	0.0084	0.056 (0.005–0.626)	0.0002
		0.0835		0.0274		0.0002
MELD score	0.936 (0.828–1.057)	0.2638	0.896 (0.786–1.022)	0.0649	0.823 (0.655–1.033)	0.0340
blood loss	1.000 (0.999–1.000)	0.3789	1.000 (0.999–1.000)	0.1649	1.000 (0.999–1.000)	0.9656
ΔA_b_	2.51e^12^ (2.364–2.67e^24^)	0.0106	4.17e^9^ (0.614–2.83e^10^)	0.0190	4.69e^13^ (5.512–3.99e^26^)	0.0048
		0.0126		0.0525		0.003

*95% CI* 95% confidence intervals

### Serial measurements of Q-CRT and ΔA_b_

**Fig. 3:**
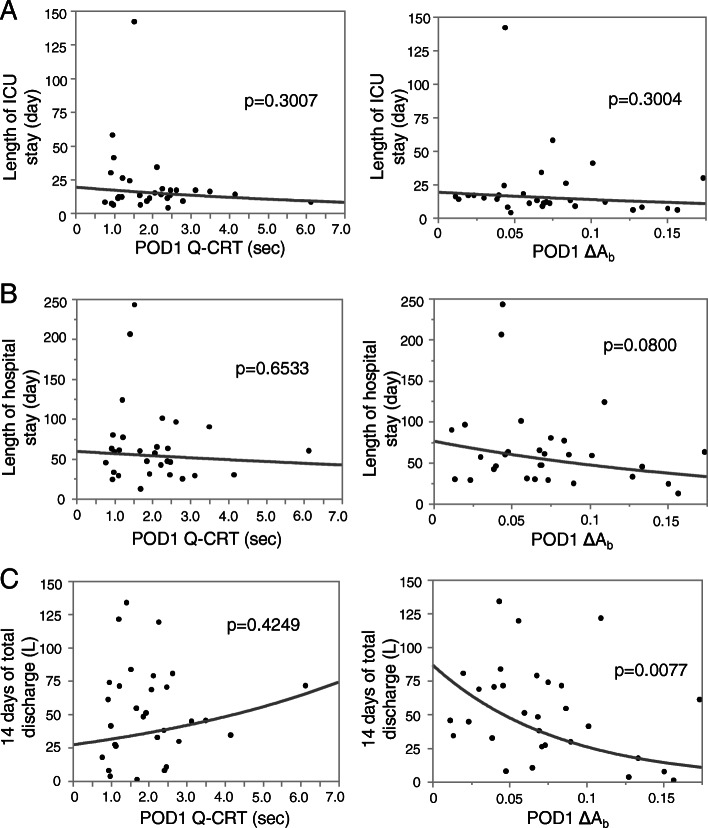
Correlations of postoperative day1 values with the outcomes. Correlations of postoperative day1 Q-CRT and ΔA_b_ with length of ICU stay (**a**), postoperative length of hospitalization (**b**), and total amount of ascites for 14 days after the surgery (**c**) are shown (*N* = 30). The *p* value is indicated in each graph

**Fig. 4:**
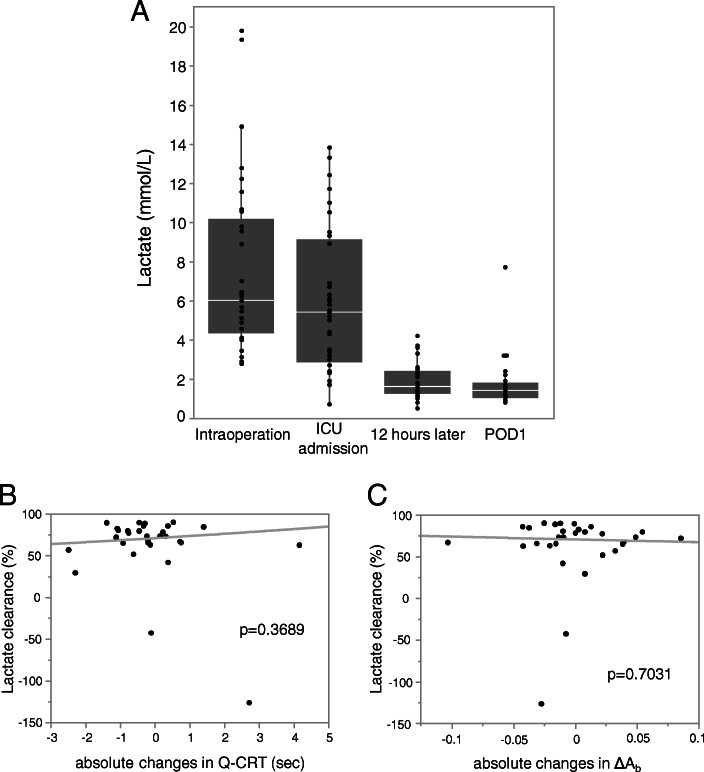
Serial measurement of lactate clearance. The lactate levels at intraoperative (maximum value), ICU admission, 12 h later, and POD1 are shown (**a**). Correlations between absolute changes in Q-CRT (**b**) and ΔA_b_ (**c**) from ICU admission to POD1 with lactate clearance (*N* = 30). The *p* value is indicated in each graph

Quantitative CRT and ΔA_b_ were measured at POD1. Due to missing data, three patients were excluded from the analysis. No significant correlations for Q-CRT at POD1 were observed between these measurements and length of ICU stay, post-surgery hospitalization, or total amount of ascites for 14 days after surgery. ΔA_b_ at POD1 did not significantly correlate with length of ICU stay or post-surgery hospitalization but with the total amount of ascitic discharge for 14 days after surgery (Fig. 3). The absolute changes in Q-CRT from ICU admission to POD1 failed to show any significant association with these outcomes. The absolute changes in ΔA_b_ from ICU admission to POD1 did not show any significant correlation with length of ICU stay or number of days of hospitalization after surgery but with the total amount of ascitic discharge for 14 days after surgery (Additional file 7). We also evaluated a possible correlation between Q-CRT and ΔA_b_ and the change of MELD score before and after the transplantation surgery. No significant correlation was found in perioperative changes of the MELD score with Q-CRT and ΔA_b_ (data not shown). Maximum lactate values during surgery, ICU admission, 12 h after admission, and POD1 are shown in Fig. 4a. No significant correlation between the absolute changes in Q-CRT and ΔA_b_ from ICU admission to POD1 with lactate clearance was observed (Fig. 4b-c). No significant correlation was observed between the lactate clearance and 14 days total discharge, length of ICU stay, and length of hospitalization after surgery (data not shown). The enrolled patients were divided into two groups by the change in Q-CRT and ΔA_b_ from ICU admission to POD1, but no significant difference in the outcomes between the two groups was observed (Table 4).

**Table 4:** Serial Q-CRT measurements; outcomes related to Q-CRT and ΔA_b_ improvement / worsening

	Q-CRT improvement (*N* = 19)	Q-CRT worsening (*N* = 11)	*p* value
Length of ICU stay (day)	12 (9–24)	14 (9–18)	0.8126
Length of hospital stay (day)	60 (33–80)	57 (30–77)	0.3891
7 days total discharge (L)	29.0 (14.5–39.6)	20.1 (12.3–32.6)	0.3776
14 days total discharge (L)	51.1 (26.2–78.9)	41.3 (29.6–71.3)	0.5612
	ΔA_b_ improvement(*N* = 13)	ΔA_b_ worsening(*N* = 17)	*p* value
Length of ICU stay (day)	17 (10–38)	13 (8–17)	0.0978
Length of hospital stay (day)	60 (30–102)	47 (32–70)	0.4896
7 days total discharge (L)	29.5 (19.7–41.1)	18.1 (8.1–32.6)	0.0824
14 days total discharge (L)	54.4 (43.0–81.2)	34.3 (14.0–70.9)	0.0753

Summary statistics are reported as medians (lower and upper quartiles). From ICU admission to POD1, those with decreased Q-CRT were defined as improved, and those with increased were defined as worsening. Those with increased delta A_b_ were defined as improved, and those with decreased were defined as worsening

## Discussion

This study evaluated tissue perfusion in patients with postoperative liver transplantations by a quantitative CRT method using a pulse oximeter. Significant associations between Q-CRT and length of ICU and hospital stays and large postoperative ascitic discharge were observed. The newly developed parameter of ΔA_b_, which is expected to reflect the total oxygen delivery to the peripheral tissues, was also significantly associated with these outcomes. These observations suggest that the newly developed Q-CRT method might be helpful to detect tissue perfusion abnormalities influencing organ function of the grafted liver in postoperative period. This suggestion may be supported by the observation that Q-CRT and ΔA_b_ were significantly correlated with portal and hepatic vein blood flow rate measured using ultrasound. Q-CRT technique is expected to be accomplished more easily than blood flow measurement using ultrasound. It should be addressed that a single measurement of peripheral tissue perfusion was associated with the outcomes; however, many related mechanisms are presumed to be involved in these significant associations. This study suggested that a simple evaluation method of Q-CRT may be useful for clinical management in liver transplantation patients, and further evaluation is absolutely necessary.

CRT is a simple and non-invasive test used to assess peripheral perfusion at the bedside. Although CRT can be used without any equipment, intra-examiner differences and poor reproducibility, even by the same observers, were reported [9, 10, 25]. CRT can be affected by the surrounding environment such as temperature and lightning [10, 26], unifying measurement condition is an important point. To overcome this problem, several investigations were conducted. Kawaguchi and colleagues developed a device that can be adjusted for pressing strength and time using an electric actuator and strength and color sensors [27, 28]. Shinozaki and colleagues used fingernail video recording and image analysis software for calculating CRT [29]. We developed a new device that can measure CRT quantitatively by using the pulse oximeter. This new device automatically compresses the finger with a constant pressure of 500 mmHg [11] and was applied to patients with suspected sepsis at ED [30]. Another study also evaluated quantitative CRT using a pulse oximeter in ED patients [31]. Our device demonstrates the advantage of measuring not only Q-CRT but ΔA_b_, an index of integrated peripheral oxygen delivery status, because pulse oximetry enables the measurement of transmitted light quantities under red and infrared light [12]. In this study, both Q-CRT and ΔA_b_ showed significant associations with post liver transplantation outcomes. Further investigation is necessary to identify which of these parameters would serve the patients better in other specific situations such as septic shock, post-cardiac surgery, and severe trauma.

Liver transplantation is expected to reduce portal hypertension and associated ascites transudation. However, massive ascites is one of the most frequent complications and poor prognostic factors in liver transplantation [32–34]. Several clinical risk factors for considerable amounts of postoperative ascites include preoperative MELD score, large blood loss volume, and small-sized grafts [35–37]. Our previous study reported that 48% of patients receiving living donor liver transplants developed massive postoperative ascites (> 1000 mL/day at POD14), and it also reported that preoperative ascites, intraoperative blood loss, and duration of anhepatic phase were associated with considerable amounts of postoperative ascites [22]. This study also disclosed that patients with a particularly high amount of preoperative ascites tended to have more postoperative ascites. The amount of ascites noted in this study was similar to our previous report [22] and other studies [33, 38]. Values and rates reflecting postoperative total bilirubin, acute rejection, and length of hospital stay were significantly higher in patients with massive ascites, although no significant impact of massive ascites on the 5-year survival rate was observed [22]. In this study, all the patients survived for the observation period of 1 year. Large amounts of diuretics including furosemide, spironolactone, and human atrial natriuretic peptide and albumin administration together with intensive evaluation of intravascular volume are required to control massive ascites.

In patients with a large amount of ascites, close monitoring of the in–out balance and optimal adjustment of fluid administration, which can be achieved in the ICU, is necessary. This may explain the correlation between the length of ICU stay and the amount of ascites present. Although management regarding ICU stay may differ in each country, several Japanese studies reported similar lengths of ICU and hospital stays to this study [39, 40]. It should be noted that other treatments, such as colloid and pressor administration, nutritional support, and antimicrobial agents against infection, should be conducted properly, in addition to a large of fluid resuscitation for perioperative management in liver transplantation.

This study demonstrated that Q-CRT and ΔA_b_ measured immediately after transplantation surgery could identify the patients who would potentially require highly intensive care due to the development of considerable postoperative ascites. Furthermore, it was shown that portal blood flow was associated with Q-CRT and ΔA_b_, and that the closer to normal range was, the less postoperative ascites. Several possible mechanisms may explain this finding. First, Q-CRT and ΔA_b_ can detect a decrease in peripheral perfusion resulting from preoperative liver failure. It is known that a reduction in systemic vascular resistance due to primary arterial vasodilatation in the splanchnic circulation is observed in patients with liver cirrhosis [41, 42]. Activated vasodilator factors such as nitric oxide [43] and endogenous cannabinoids [44] before surgery might exhibit some impact even immediately after transplantation surgery on peripheral tissue perfusion. Second, Q-CRT and ΔA_b_ may indicate the progress of normalization of portal blood flow, and detect the reduced perfusion caused by endotoxin. It is recognized that portal vein blood flow is normalized as the function of the graft liver is restored [45]. A high portal pressure is strongly associated with poor liver transplantation outcomes [46, 47]. A high portal venous pressure reportedly increases gut-related bacteremia in living donor patients with liver transplantations [48] and increases blood endotoxin levels [49, 50]. Several mechanisms such as a vulnerable intestinal barrier [51], changes in the intestinal microbiota [52], and decreased endotoxin clearance in the liver [53] were reported. Hepatic ischemia reperfusion injury negatively affects allograft function following liver transplantation. Although many different involved cell types (sinusoidal endothelial cells, hepatocytes, Kupffer cells, neutrophils, and platelets) and mechanisms (toll-like receptor signaling, micro-RNA expression, production of reactive oxygen species, autophagy, and activation of hypoxia-inducible factors) were reported so far [54], it is uncertain whether Q-CRT can detect hepatic ischemia reperfusion injury. It should be noted that many pathological mechanisms, other than ischemia reperfusion injury, are deemed to contribute to poor outcomes for liver transplantation.

Elevated blood lactate levels traditionally were considered a signal of tissue hypoxia. The third international consensus definitions for sepsis and septic shock (Sepsis-3) defines septic shock as a clinical construct of sepsis with persisting hypotension requiring vasopressors to maintain a MAP ≥65 mmHg and exhibiting a serum lactate level > 2 mmol/L despite adequate volume resuscitation [55]. Our earlier study found that ΔA_b_ was significantly associated with high lactate levels [12], and we observed a significant correlation between Q-CRT and ΔA_b_ and mean artery pressure in this study, although we did not find any correlation between Q-CRT and ΔA_b_ with the blood lactate level. The liver plays a major role in systemic lactate clearance, and hyperlactatemia is related to impaired hepatic clearance in advanced cirrhosis [56]. The patients in this study experienced an anhepatic period of approximately 2 h duration. It assumed that blood lactate and Q-CRT and ΔA_b_ were reflecting different conditions of the patients immediately following liver transplantation surgery; elevated blood lactate was largely influenced by insufficient liver function, and Q-CRT and ΔA_b_ were reflective of peripheral perfusion. The lactate value decreased following perfusion, supporting the lactate clearance function in the liver.

In contrast to Q-CRT and ΔA_b_ values measured immediately after surgery, those measured at POD1 were not significantly associated with the outcomes. Changes from immediately post- surgery to POD1 demonstrated no significant association; however, serial measurements would have provided more information than just single measurements. Several clinical interventions, such as fluid administration, optimizing sedation depth, and mechanical ventilation settings with spontaneous breathing, may affect Q-CRT and ΔA_b_ measurements. It is known that a reliable analysis of respiratory changes in arterial pressure is possible in patients who are sedated and mechanically ventilated with conventional tidal volumes [57, 58]. Intraoperative measurements may provide a more accurate prediction of postoperative outcomes, as hemodynamics, sedative status, and ventilation are more homogenously managed during general anesthesia. Further assessment during surgery is necessary in order to demonstrate this conjecture.

Several limitations that might affect the results of this study should be acknowledged. First, this study was performed at a single ICU, and the number of patients was relatively small. Second, no evaluation study of Q-CRT and ΔA_b_ using the widely accepted gold standard of hemodynamic assessment such as cardiac output, oxygen delivery (DO_2_) and oxygen consumption (VO_2_) has been conducted to date. Third, Q-CRT and ΔA_b_ measurements were recorded only at ICU admission and POD1. As discussed above, adding intraoperative and preoperative measurements may have provided more useful information. Presumably, the hypoperfusion evaluated by Q-CRT and ΔA_b_ measurement reflects hypoperfusion of the grafted liver, and hypoperfusion to the liver might have induced massive ascites. Further investigation is necessary to clarify the pathophysiological mechanism causing massive ascites after liver transplantation. Finally, an earlier study reported CRT longer than 3 s was not predictive of mortality in patients complicated with malarial anemia [59], although no patients manifested severe anemia in our- study.

## Conclusion

This prospective observational study found that Q-CRT with a pulse oximeter was significantly associated with postoperative outcomes in patients receiving liver transplantations. Q-CRT may be relevant as a new non-invasive monitoring tool for patients treated in ICUs upon validation by several different ICU patient cohorts such as post-cardiac surgery, heart failure, and patients with multiple organ failures.

## Supplementary information

**Additional file 1.** Preoperative baseline characteristics, graft conditions, surgery-related factors, clinical parameters at ICU admission after surgery.

**Additional file 2 **Correlations of Q-CRT and ΔA_b_ with clinical parameters. Correlations of Q-CRT and ΔA_b_ with heart rate (HR) (A), central venous pressure (CVP) (B), blood lactate (C), hemoglobin (Hb) (D), hepatic arterial velocity (HA) (E), hepatic venous velocity (HV) (F) are shown Measures observed at similar time points (*N* = 33). The *p* value is indicated in each graph.

**Additional file 3 **Correlations of liver blood flow with the outcomes. Correlation with the total amount of discharge for 7 days and 14 days post-surgery with PV (A) and HA (B) and HV (C) are shown (*N* = 33). The *p* value is indicated in each graph.

**Additional file 4 **Postoperative ascites in relation to preoperative ascites. Postoperative total amounts of discharge at POD7 (A) and POD14 (B) in the none (no ascites, *N* = 20), the non-massive (1–999 ml, *N* = 4), and the massive (more than 1000 ml, *N* = 9) groups were shown. The *p* value is indicated in each graph.

**Additional file 5.** Comparison of the non-massive ascites group with the massive ascites group.

**Additional file 6.** The cut-off values of Q-CRT and ΔA_b_ for each outcome.

**Additional file 7 **Serial measurements of quantitative capillary refill time and ΔA_b_. Correlations between the absolute changes in Q-CRT and ΔA_b_ from ICU admission to POD1 with ICU stay (A), postoperative length of hospitalization (B), and total amount of ascites for 14 days after the surgery (C) are shown (*N* = 30). The *p* value is indicated in each graph.

## Data Availability

The datasets used and/or analyzed during the current study available from the corresponding author on reasonable request.

## Abbreviations

AUC: area under the curve
CI: confidence interval
CRT: capillary refill time
CVP: central venous pressure
ED: emergency department
HA: hepatic artery
Hb: hemoglobin
HR: heart rate
HV: hepatic vein
ICU: intensive care unit
MAP: mean arterial pressure
MELD: model for end-stage liver disease
POD: postoperative day
PV: portal vein
PT-INR: prothrombin time international normalized ratio
Q-CRT: quantitative capillary refill time
ROC: receiver operating characteristic curve
ΔA_b_: delta A_b_
