# Supplementary material for: Pulse oximetry-based capillary refilling evaluation predicts postoperative outcomes in liver transplantation: a prospective observational cohort study
Source: BMC Anesthesiol. 2020 Sep 29;20:251. doi: 10.1186/s12871-020-01171-y (PMC7523076; doi:10.1186/s12871-020-01171-y)
Supplement: Supplementary file 1 — Additional file 1. Preoperative baseline characteristics, graft conditions, surgery-related factors, clinical parameters at ICU admission after surgery. [file 12871_2020_1171_MOESM1_ESM.pdf]

Additional file 1. Preoperative baseline characteristics, graft conditions, surgery-related factors, clinical parameters at ICU admission after surgery.

Variables		
Recipient characteristics	body mass index (kg/m²)	21.0 (19.0–24.4)
	primary disease	
	HBV positive	2 (6.1%)
	HCV positive	3 (9.1%)
	autoimmune disease (PBC/PSC/AIH)	8 (24.2%)
	alcoholic	5 (15.2%)
	others	15 (45.5%)
	albumin (g/dL)	2.8 (2.4–3.2)
	total bilirubin (mg/dL)	2.3 (1.2–7.4)
	PT-INR	1.27 (1.10–1.57)
Graft characteristics	living donor	27 (81.8%)
	age (years)	43 (32–54)
	male sex	8 (27.6%)
	graft volume vs. recipient's SLV (%)	
	living donor	44.2 (38.0–51.1)
	brain death donor	114.0 (95.0–140.1)
	GRWR (%)	
	living donor	0.89 (0.70–1.06)
	brain death donor	2.29 (1.84–2.70)
Operative factors	operating time (hour)	10.4 (9.4–11.6)
	anhepatic phase (min)	148 (137–194)
	graft ischemic time (min)	140 (127–156)
	blood loss (mL)	3640 (1835–6743)
	blood transfusion (ml)	3000 (1600–5880)
	intraoperative fluid balance (mL)	3810 (2780–4978)
Clinical parameters at ICU admission	body weight (kg)	58.7 (54.6–70.3)
	pulmonary arterial systolic pressure (mmHg)	32 (25–39)
	central venous pressure (cmH_2O)	12 (9–15)
	central venous oxygen saturation (%) *	85 (84–89)
	pulse pressure variation (%) **	4 (3–6)
	hemoglobin (mg/dL)	8.8 (8.2–9.7)

	hepatic arterial flow velocity (cm/sec)	42.5 (31.7–66.5)
	hepatic venous flow velocity (cm/sec)	36.8 (27.4–46.0)

*N=26, **N=30

Summary statistics are reported as No. (%), medians (lower and upper quartiles).

PBC; primary biliary cirrhosis, PSC; primary sclerosing cholangitis, AIH; autoimmune hepatitis, SLV; standard liver volume, GRWR; graft to recipient weight ratio
