# Supplementary figures and images for: Pulse oximetry-based capillary refilling evaluation predicts postoperative outcomes in liver transplantation: a prospective observational cohort study
Source: BMC Anesthesiol. 2020 Sep 29;20:251. doi: 10.1186/s12871-020-01171-y (PMC7523076; doi:10.1186/s12871-020-01171-y)

## Additional file 2.

Correlations of Q-CRT and  $\Delta A_b$  with clinical parameters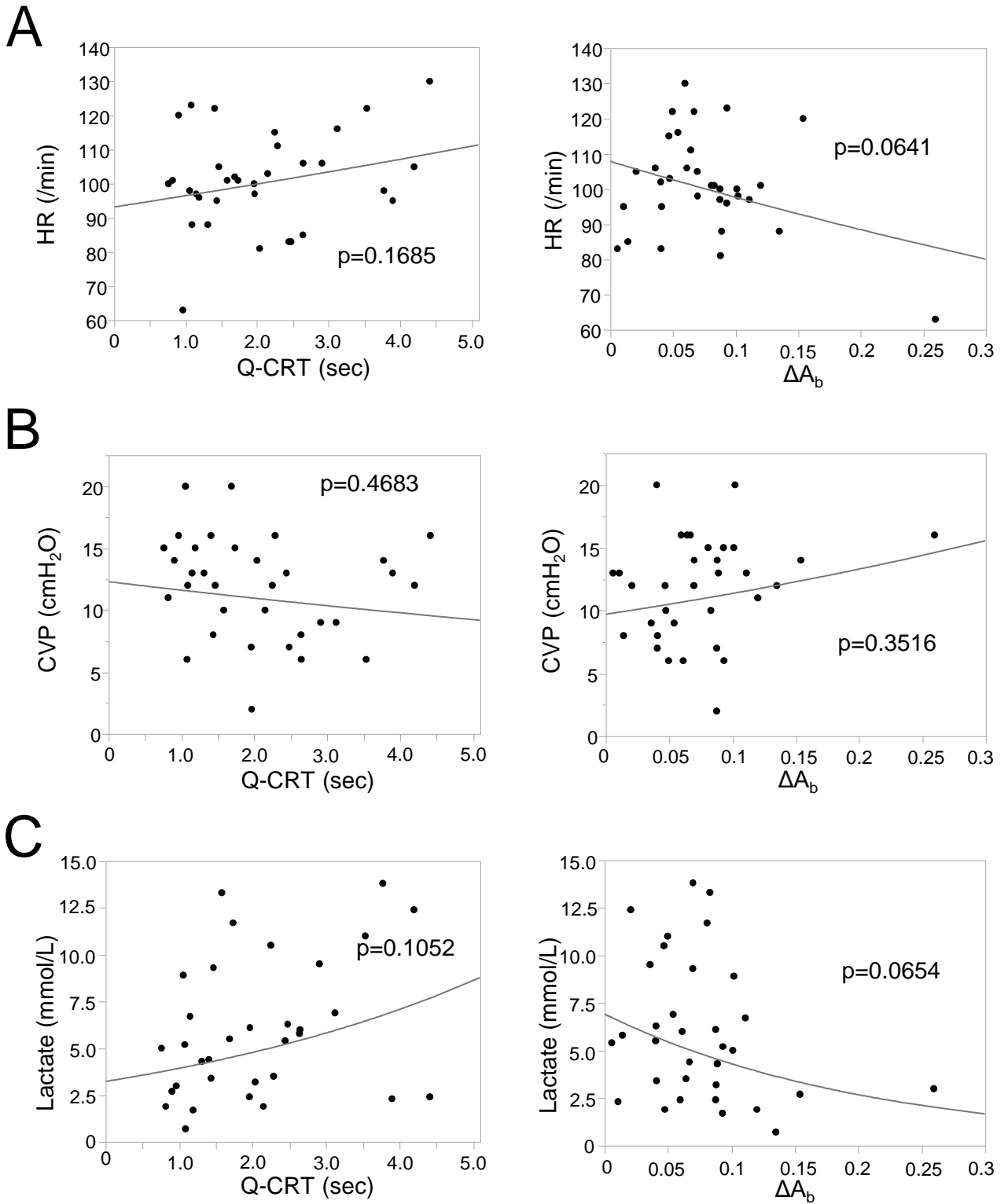

D

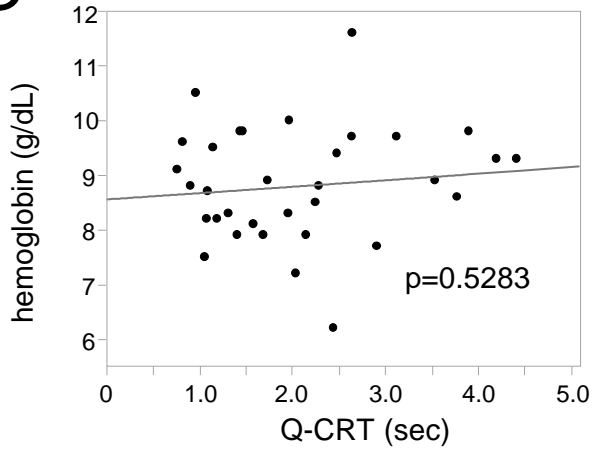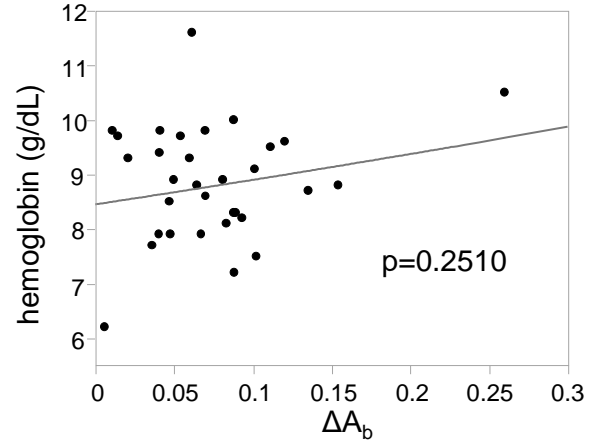

E

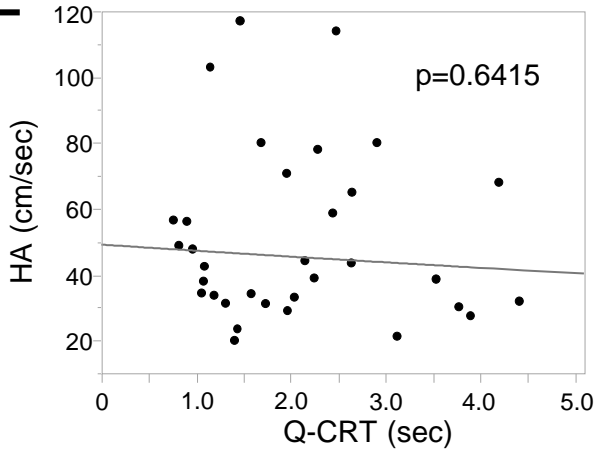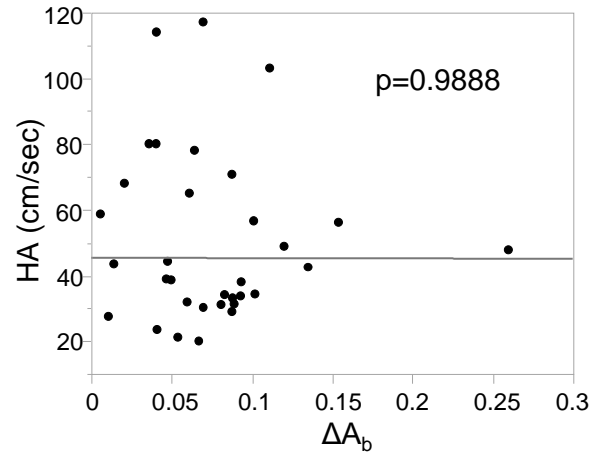

F

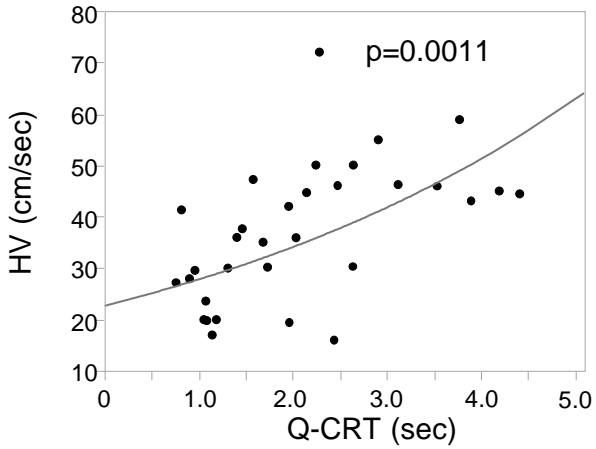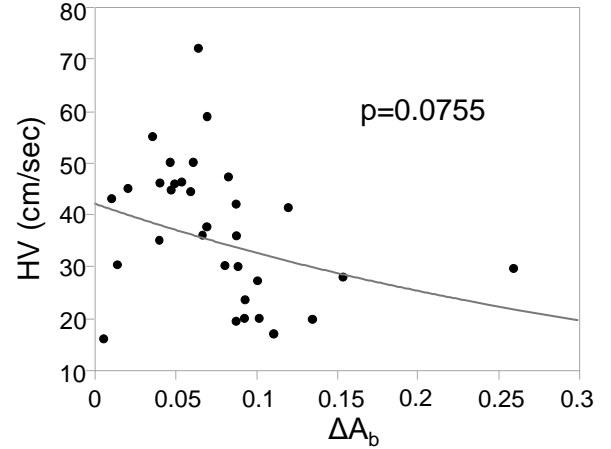

Supplement: Supplementary file 2 — Additional file 2 Correlations of Q-CRT and ΔAb with clinical parameters. Correlations of Q-CRT and ΔAb with heart rate (HR) (A), central venous pressure (CVP) (B), blood lactate (C), hemoglobin (Hb) (D), hepatic arterial velocity (HA) (E), hepatic venous velocity (HV) (F) are shown Measures observed at similar time points (N = 33). The p value is indicated in each graph. [file 12871_2020_1171_MOESM2_ESM.pdf]

### Additional file 3.

### Correlations of liver blood flow with the outcomes

**A**

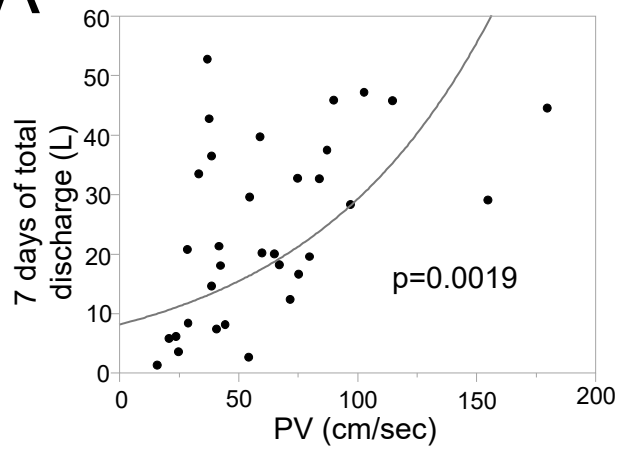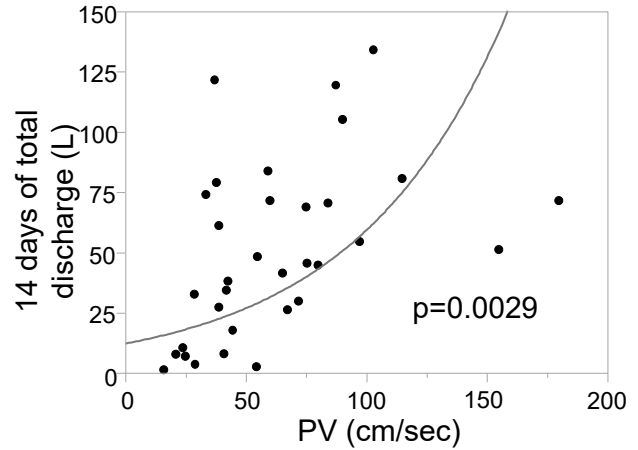

**B**

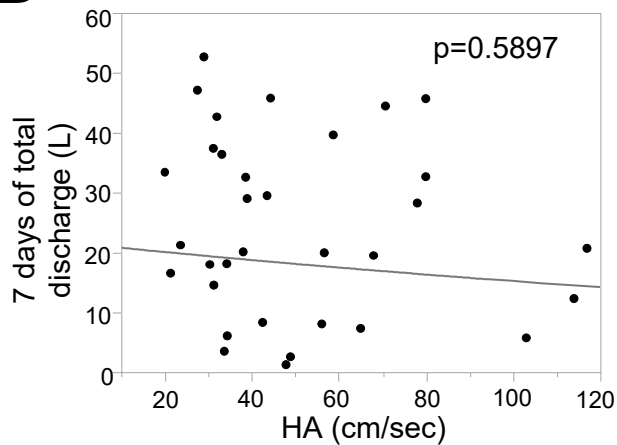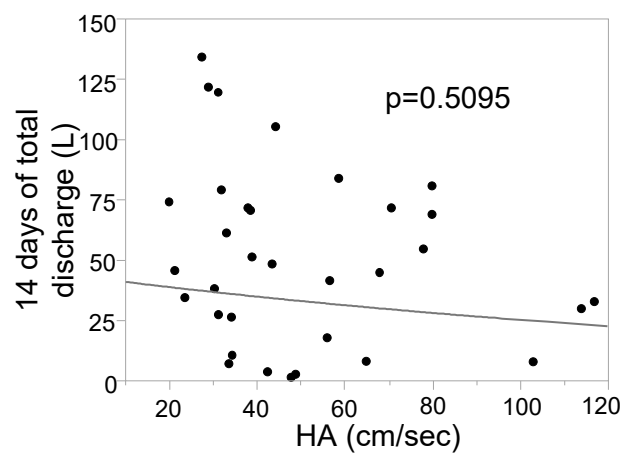

**C**

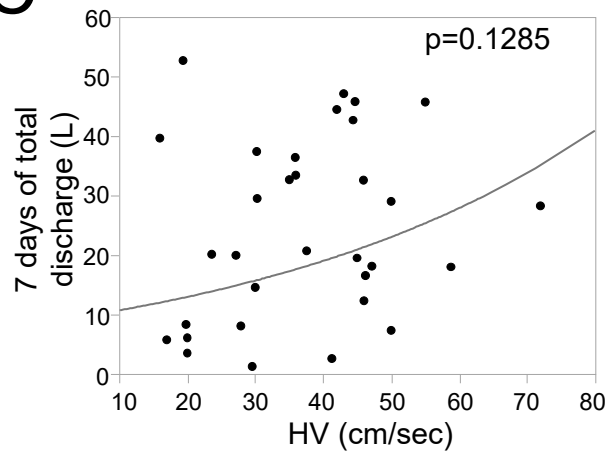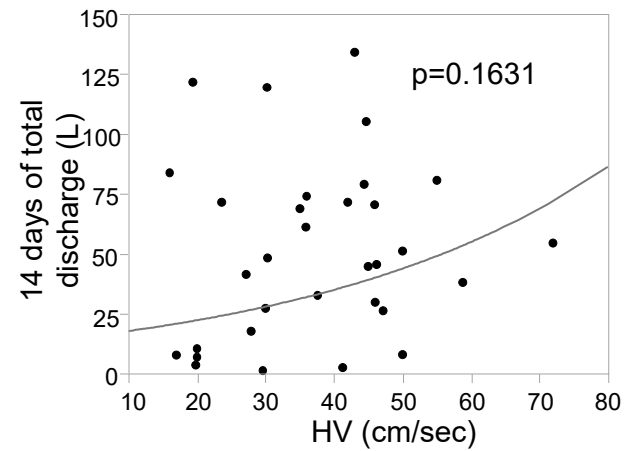

Supplement: Supplementary file 3 — Additional file 3 Correlations of liver blood flow with the outcomes. Correlation with the total amount of discharge for 7 days and 14 days post-surgery with PV (A) and HA (B) and HV (C) are shown (N = 33). The p value is indicated in each graph. [file 12871_2020_1171_MOESM3_ESM.pdf]

## Additional file 4.

### Postoperative ascites in relation to preoperative ascites

**A**

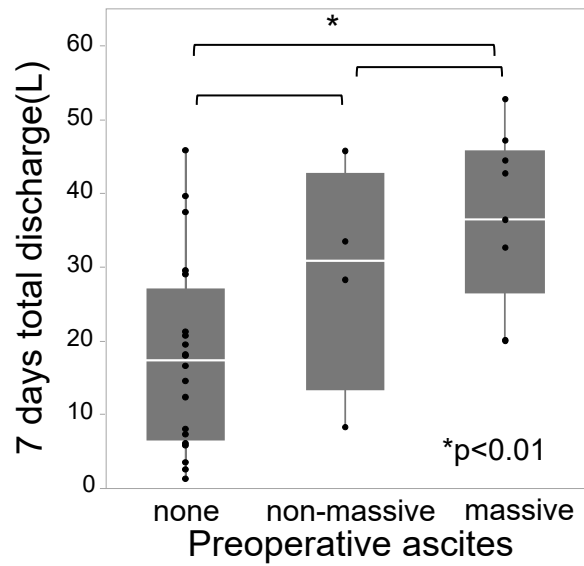

**B**

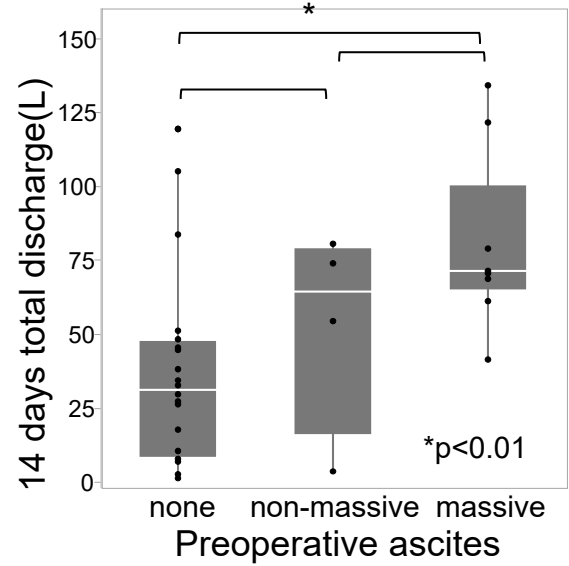

Supplement: Supplementary file 4 — Additional file 4 Postoperative ascites in relation to preoperative ascites. Postoperative total amounts of discharge at POD7 (A) and POD14 (B) in the none (no ascites, N = 20), the non-massive (1–999 ml, N = 4), and the massive (more than 1000 ml, N = 9) groups were shown. The p value is indicated in each graph. [file 12871_2020_1171_MOESM4_ESM.pdf]

Additional file 7.

Serial measurements of quantitative capillary refill time and  $\Delta A_b$

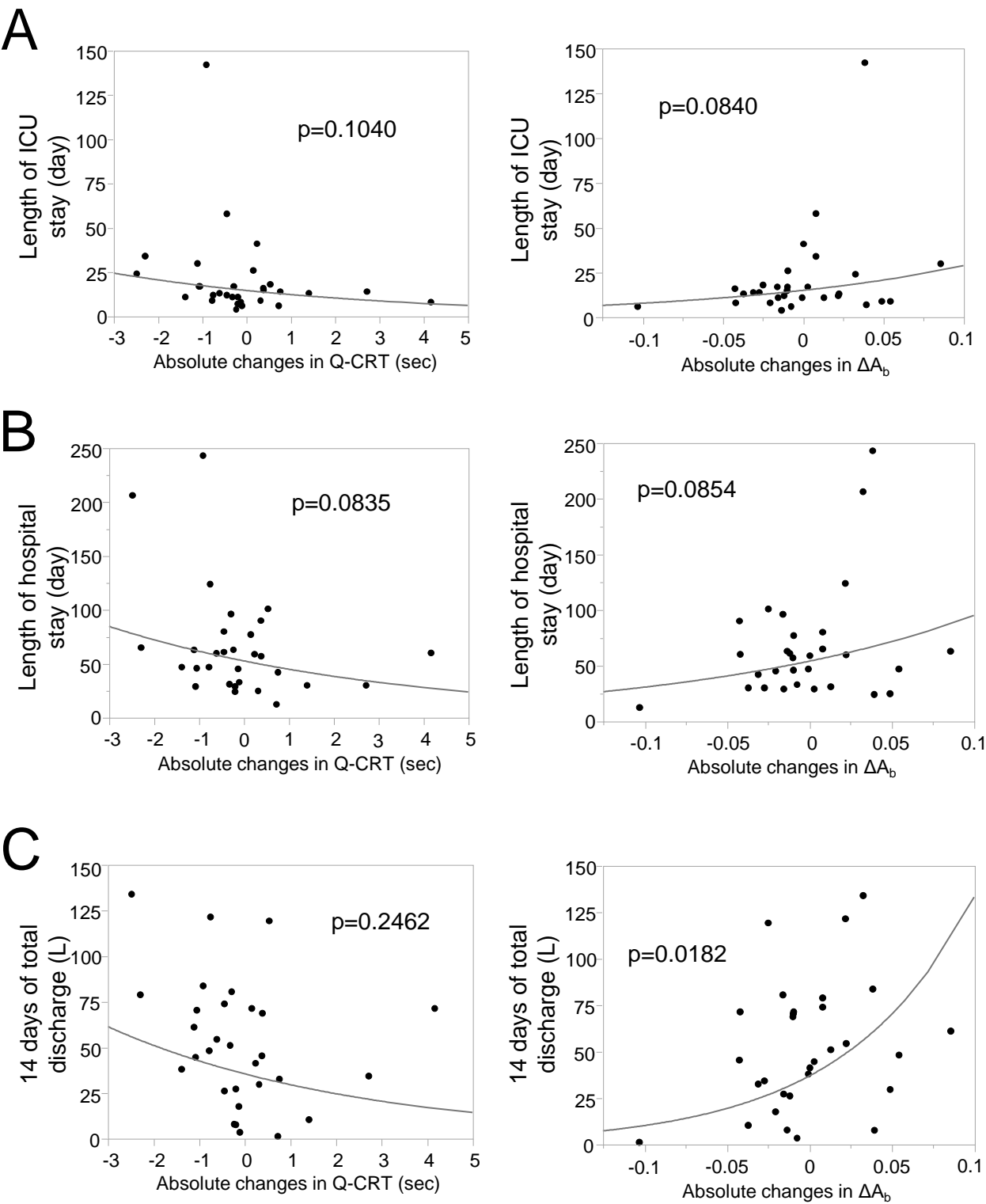

Supplement: Supplementary file 7 — Additional file 7 Serial measurements of quantitative capillary refill time and ΔAb. Correlations between the absolute changes in Q-CRT and ΔAb from ICU admission to POD1 with ICU stay (A), postoperative length of hospitalization (B), and total amount of ascites for 14 days after the surgery (C) are shown (N = 30). The p value is indicated in each graph. [file 12871_2020_1171_MOESM7_ESM.pdf]
