# Supplementary material for: Pulse oximetry-based capillary refilling evaluation predicts postoperative outcomes in liver transplantation: a prospective observational cohort study
Source: BMC Anesthesiol. 2020 Sep 29;20:251. doi: 10.1186/s12871-020-01171-y (PMC7523076; doi:10.1186/s12871-020-01171-y)
Supplement: Supplementary file 5 — Additional file 5. Comparison of the non-massive ascites group with the massive ascites group. [file 12871_2020_1171_MOESM5_ESM.pdf]

Additional file 5. Comparison of the non-massive ascites group with the massive ascites group.

		Non-massive ascites group (N=10)	Massive ascites group (N=23)	p value
Recipient characteristics	body mass index (kg/m²)	23.2 (20.9–25.7)	20.2 (18.9–24.2)	0.0379
	primary disease			1.0000
	HBV positive	1 (3.0%)	1 (3.0%)	
	HCV positive	1 (3.0%)	2 (6.1%)	
	autoimmune disease (PBC/PSC/AIH)	2 (6.1%)	6 (18.2%)	
	alcoholic	1 (3.0%)	4 (12.1%)	
	others	5 (15.2%)	10 (30.3%)	
	albumin (g/dL)	2.7 (2.3–3.0)	2.8 (2.4–3.3)	0.3769
	total bilirubin (mg/dL)	1.9 (0.6–3.8)	2.5 (1.3–14.0)	0.1080
	PT-INR	1.11 (1.00–1.44)	1.28 (1.15–1.67)	0.0684
Graft characteristics	living donor	6 (60%)	21 (91.3%)	0.0321
	age (years)	35 (31–58)	45 (31–53)	0.8984
	male sex	1 (14.3%)	7 (31.8%)	0.3660
	graft volume vs. recipient's SLV (%)			
	living donor	49.5 (36.8–50.1)	42.0 (35.5–51.5)	0.7706
	brain death donor	114.0 (98.3–127.4)	132.9 (94.8–171.0)	1.0000
	GRWR (%)			
	living donor	0.98 (0.64–1.03)	0.82 (0.73–1.09)	0.9071
	brain death donor	2.88 (1.87–2.46)	2.60 (1.87–3.32)	0.6434
Operative factors	operating time (hrs)	10.1 (8.8–12.4)	10.4 (9.6–11.5)	0.7244
	anhepatic phase (min)	145 (132–172)	149 (139–199)	0.4046
	graft ischemic time (min)	132 (124–159)	141 (129–156)	0.5193
	blood loss (mL)	2210 (1625–4005)	3845 (2330–8160)	0.0848
	blood transfusion (ml)	1940 (750-3300)	3440 (1680-6240)	0.0600
	intraoperative fluid balance (mL)	3523 (2809–5208)	3907 (1932–4960)	0.9064

Clinical	body weight (kg)	69.9 (58.2–74.6)	57.8 (50.7–66.0)	0.0379
parameters at	central venous pressure (cmH_2O)	12 (10–15)	13 (8–15)	0.8905
ICU admission	hemoglobin (mg/dL)	9.6 (8.2–10.0)	8.8 (7.9–9.3)	0.1414
Outcomes	day14 discharge (mL)	0 (0–504)	3219 (2301–4175)	<0.0001

Summary statistics are reported as No. (%), medians (lower and upper quartiles).

PBC; primary biliary cirrhosis, PSC; primary sclerosing cholangitis, AIH; autoimmune hepatitis, SLV; standard liver volume, GRWR; graft to recipient weight ratio
