# Supplementary material for: Pulse oximetry-based capillary refilling evaluation predicts postoperative outcomes in liver transplantation: a prospective observational cohort study
Source: BMC Anesthesiol. 2020 Sep 29;20:251. doi: 10.1186/s12871-020-01171-y (PMC7523076; doi:10.1186/s12871-020-01171-y)
Supplement: Supplementary file 6 — Additional file 6. The cut-off values of Q-CRT and ΔAb for each outcome. [file 12871_2020_1171_MOESM6_ESM.pdf]

Additional file 6. The cut-off values of Q-CRT and ΔA_b for each outcome

	Q-CRT				ΔA_b			
	AUC (95% CI)	cut off value	sensitivity	specificity	AUC (95% CI)	cut off value	sensitivity	specificity
Length of ICU stay (day)	0.930 (0.777–0.981)	1.964	0.87	1.00	0.870 (0.684–0.954)	0.0697	0.83	0.80
Length of hospital stay (day)	0.896 (0.688–0.971)	2.478	0.87	0.80	0.857 (0.652–0.950)	0.0473	0.83	0.80
7 days total discharge (L)	0.935 (0.796–0.981)	1.406	0.83	1.00	0.909 (0.748–0.971)	0.0401	0.74	1.00
14 days total discharge (L)	0.974 (0.876–0.995)	0.760	0.87	1.00	0.965 (0.855–0.992)	0.0697	0.87	1.00

95% CI, 95% confidence interval
